# Two propeller flaps in a distal lower leg with bilateral defects as a single-stage procedure: A case report

**DOI:** 10.1016/j.jpra.2025.03.006

**Published:** 2025-03-19

**Authors:** Adam Stepniewski, Alperen Sabri Bingoel, Katharina Jäckle, Wolfgang Lehmann, Gunther Felmerer

**Affiliations:** a Division of Plastic Surgery, Department for Trauma Surgery, Orthopedics and Plastic Surgery, University Medical Center Göttingen, 37075 Göttingen, Germany; b Department for Trauma Surgery, Orthopedics and Plastic Surgery, University Medical Center Göttingen, 37075 Göttingen, Germany

**Keywords:** Propeller flap, Free flap, Lower leg, Soft tissue defect, Ankle fracture, Reconstruction

## Abstract

Soft tissue defects in the lower leg often represent a therapeutic challenge. One of the treatment modalities is plastic reconstruction with a propeller flap. In our case report, we would like to present a case of a 75-year-old, multimorbid female patient who developed in the right distal lower leg, both medial and lateral, soft tissue defects after open reduction and internal fixation of a complex ankle fracture. She was treated by the single surgeon with two propeller flaps, one lateral and one medial, in the same operation.

With this single-stage procedure, we achieved effective, long-term closure of the defect. It resulted in several advantages for the patient, including a shorter operation time compared to hypothetic two free flaps and no additional prolonged anesthesia. Also, defects were reconstructed according to the “like with like” principle, and less donor site morbidity compared to free muscle flaps. The disadvantage of the procedure was the need for split skin graft of the donor sites. Based on our experience, this method could be an option for carefully selected patients without high aesthetical demands who have undergone appropriate preoperative systemic and local optimization.

## Introduction

One of the etiologies of soft tissue defects in the lower leg are postoperative complications. Surgeons can choose from various therapeutic methods with their advantages and disadvantages. The concept of the reconstructive supermarket offers optimal results not only in terms of aesthetics, but above all in terms of function.^1^ In this case report, we would like to present a patient with a complex ankle fracture accompanied by the presence of numerous pre-existing conditions, in which two large soft tissue defects in the distal lower leg, both medial and lateral, were successfully treated by the same surgeon using two propeller flaps in one operation.

## Case report

A 75-year-old female patient was admitted after a fall with a closed, second-degree trimalleolar fracture AO-type 44 B3 of the right ankle. Several pre-existing conditions were known including osteoporosis, incipient dementia, arterial hypertension, bronchial asthma, hypothyroidism, intermittent atrial fibrillation with therapeutic anticoagulation, epilepsy, cerebral infarction in the past. After initial treatment with immobilization in a plaster cast, subsequent change of anticoagulation to heparin and reduction of swelling, the patient was operated five days later using an open reduction and internal fixation (ORIF). Postoperatively, there was a wound healing delay medially with subsequent soft tissue defect, which led to debridement and vacuum assisted closure (V.A.C.®) eight days later. The intraoperative microbiological findings showed tissue contamination with *Staphylococcus epidermidis*, resistant to clindamycin and erythromycin. A computed tomography angiography (CTA) revealed a two-vessel run-off on the right lower leg with chronic occlusion of the posterior tibial artery starting right below the popliteal trifurcation. In addition, a wound healing delay with soft tissue defect also occurred laterally. After further medial and lateral debridement, a percutaneous transluminal angioplasty (PTA) was performed with recanalization of the posterior tibial artery down to the level of the middle tibia. Complete recanalization down to the foot was not possible. Four days later, the wounds were revised again with additional removal of the implants, application of the external fixator and V.A.C.®. The findings were presented to the division of plastic surgery, where a reconstruction with propeller flaps was suggested.

The patient consented both, lateral and medial reconstruction. Initially only one flap per operation was planned, first laterally and then medially. The corresponding operation took place nine days after the last debridement. First, the fixator was temporarily and partially removed laterally in the way not jeopardizing joint stability and the reduction. Radical debridement was then performed. As the next step, a perforator-pedicled, originating from peroneal artery, 170-degree rotated propeller flap was performed in the usual manner. Direct closure of the donor site with sutures was only partially possible, which is why a split-thickness skin graft from the thigh was used. The flap and its donor site were sealed with V.A.C.® and the fixator was reattached. As the patient was stable during the time up to this point and her vital parameters were all within the normal range, the decision was made to also reconstruct the medial defect in the same session which was performed as on the lateral side with a perforator-pedicled, originating from posterior tibial artery, 180-degree rotated propeller flap. The entire surgery took 438 min.

Several days postoperatively, a crescent-shaped, approx. 5 cm^2^ superficial epidermal necrosis appeared at the distal end of the medial flap, which healed successfully by secondary intention.

**Figure 1:**
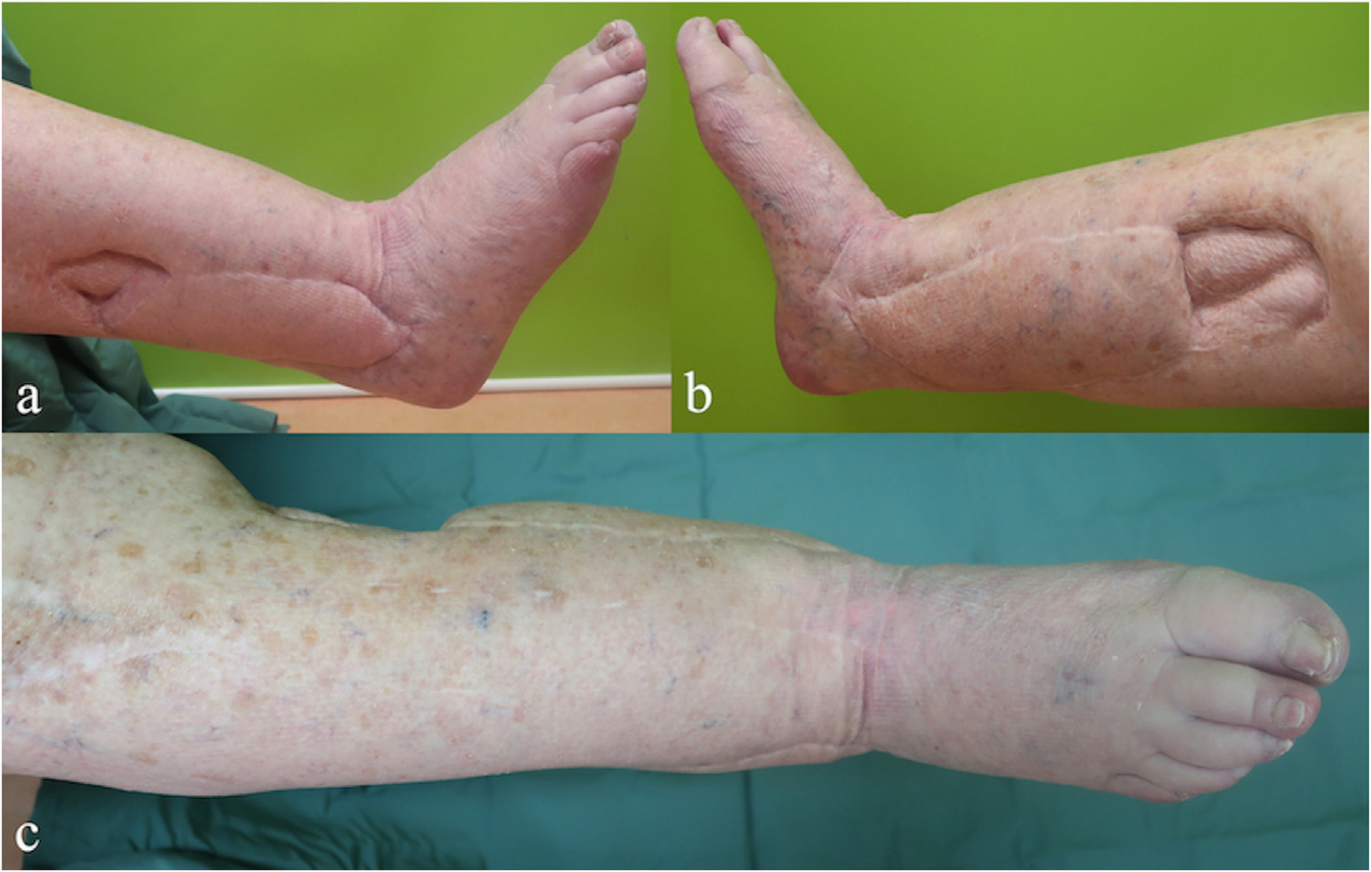
View on lateral propeller flap (a), medial propeller flap (b) and both sites from anterior (c) four years after surgery.

The patient underwent two more operations during the same hospitalization, i.e. the external fixator was removed. Eight days later, after confirmation of sterile microbiological findings, a trans-calcaneal, retrograde nail arthrodesis was performed as a permanent solution. The patient was discharged with intact and completely vital integument. She was last examined in our outpatient clinic four years after the last surgery. Local findings at the right lower leg remained intact (Figure 1).

## Discussion

Propeller flaps have become increasingly popular, particularly in the last three decades.^2^ However, despite the worldwide use of propeller flaps for over 30 years, we were unable to identify any publication that described a similar procedure performed by a single surgeon using two propeller flaps on the same lower leg in one operation.

Free flaps require similar surgical skills compared to propeller flaps, but are more complex and difficult to perform.^3^ Experience shows that they provide equally good results as free flaps but result in a reduced operation time and shorter hospitalization.^3^^,^^4^ Our operation took 438 min, but the initial debridement of the defects, the removal and reattachment of the fixator on each side separately took approx. 90 min of the surgery time. This means that the actual flap coverage took an average of 174 min per side. This time is consistent with experience from surgeons of other clinical centers. Hypothetically, a parallel operation by two teams lateral and medial would have shortened that time by half, but it would have made the complete dismounting of the fixator necessary. This intervention was not possible due to the risk of loss of reduction. Furthermore, free flap would have meant two separate operations for the patient, which together would have taken up to 600 min, unless, e.g., a single free flap with two separate skin islands or deepithelialized area and tunneling had been performed.

Propeller flaps cause lower functional losses at the donor site compared to free muscle flaps, which are 57.5 % of free flaps performed at the lower leg, but have higher donor site morbidity than free fasciocutaneous flaps harvested from healthy, non-traumatized area.^4^ They offer reconstruction according to the principle of “like with like”, however, one of the conditions is the intact tissue surrounding the defect. They also make it possible to avoid microsurgical anastomoses with main vessels.^3^ This reduces the risk of further circulatory deterioration in an impaired vascular system of the lower leg due to steal effect.

Propeller flaps also allow reconstruction of large defects up to 200 cm^2^.^5^^,^^6^ However, some authors recommend them only for small and medium-sized defects whereas large defects should be reconstructed with free perforator flaps.^7^ In 30–67 % of cases, direct closure of the donor site by direct suturing is not possible. In such cases, however, a split-thickness skin graft is necessary, thus downgrading the aesthetical outcome.^3^^,^^5^ Although the complication rate is similar to that of free flaps, local partial necrosis of varying depths appears to be over twice as high in propeller flaps, particularly in the distal area (6.88 % vs. 2.70 % in free flaps).^4^ Regarding risk factors, the literature contains varying information. As main risk factors, some authors cite patient age over 60 years, manifest diabetes and angiopathies, while others consider as the only risk factor defect localization in the distal third of the lower leg.^3^^,^^5^ To lower the risk of complications, we recommend preoperative improvement of limb perfusion. We routinely perform CTA in our high-risk patients and, if necessary, a PTA.

## Summary

In our opinion, the decision in favor of the single-stage procedure was justified by the long-term success of the treatment. Based on our experience, this method could be an option for carefully selected patients without high aesthetical demands who have undergone appropriate preoperative systemic and local optimization, implying that the treatment decision is not always easy and must always be made on an individual basis.

## Conflict of interest

All authors declare that they have no conflict of interest.

## Appendix Supplementary materials

Figure 2. Radiological findings after ORIF: anterior view (a), oblique view (b), lateral view (c). Image, image 1

Figure 3. View on lateral defect (a) and after reconstruction with propeller flap (b); view on medial defect (c) and after reconstruction with propeller flap (d). Image, image 2

Figure 4. Radiological findings after arthrodesis with retrograde intra-medullar nail (Valor™, Hindfoot Fusion Nail System, Wright Medical Group N.V.): anterior view (a), lateral view (b). Image, image 3
