# Supplementary figures and images for: Two propeller flaps in a distal lower leg with bilateral defects as a single-stage procedure: A case report
Source: JPRAS Open. 2025 Mar 19;44:212–6. doi: 10.1016/j.jpra.2025.03.006 (PMC11993176; doi:10.1016/j.jpra.2025.03.006)

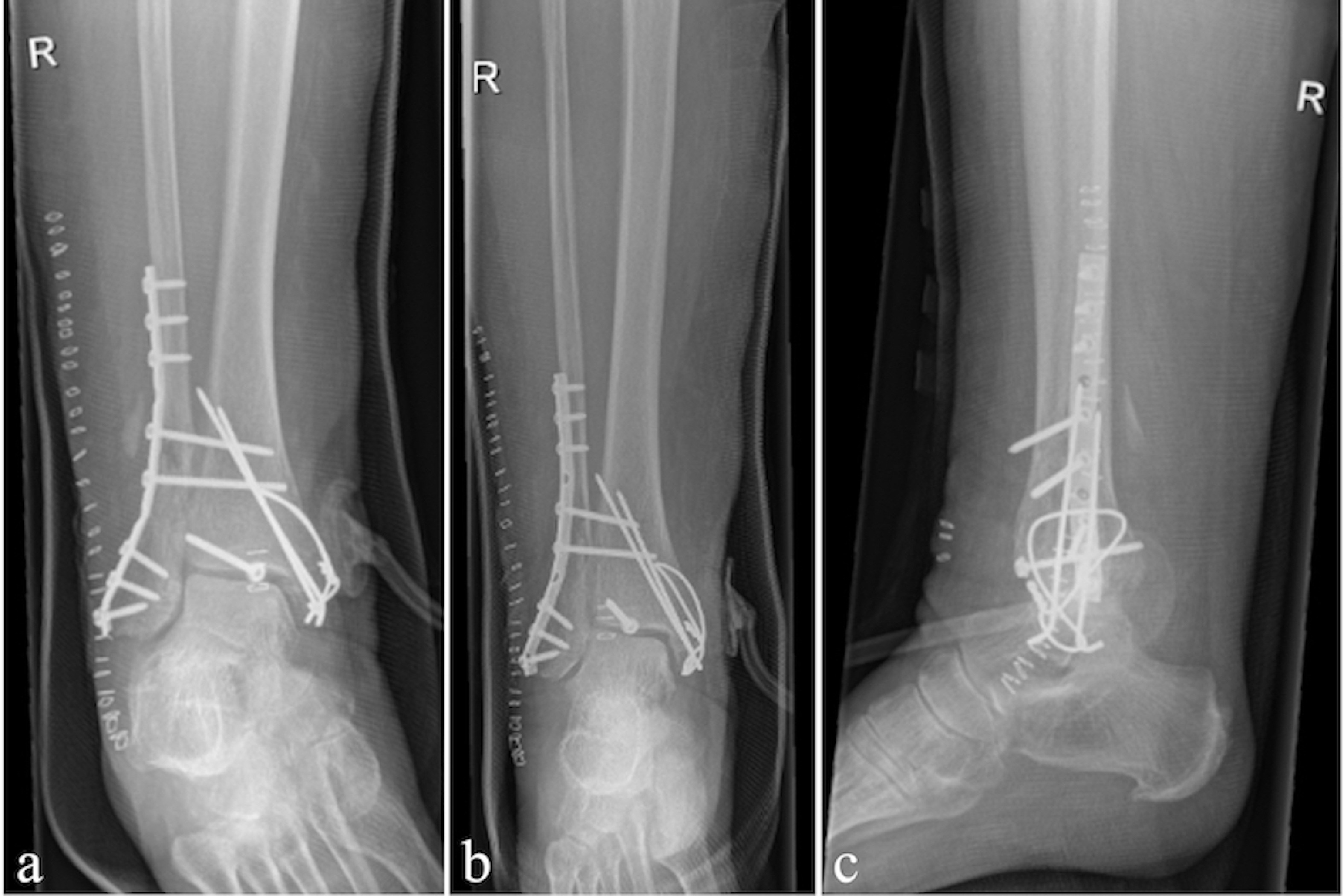

Supplement: Supplementary file 1 — Figure 2. Radiological findings after ORIF: anterior view (a), oblique view (b), lateral view (c). [file mmc1.jpg]

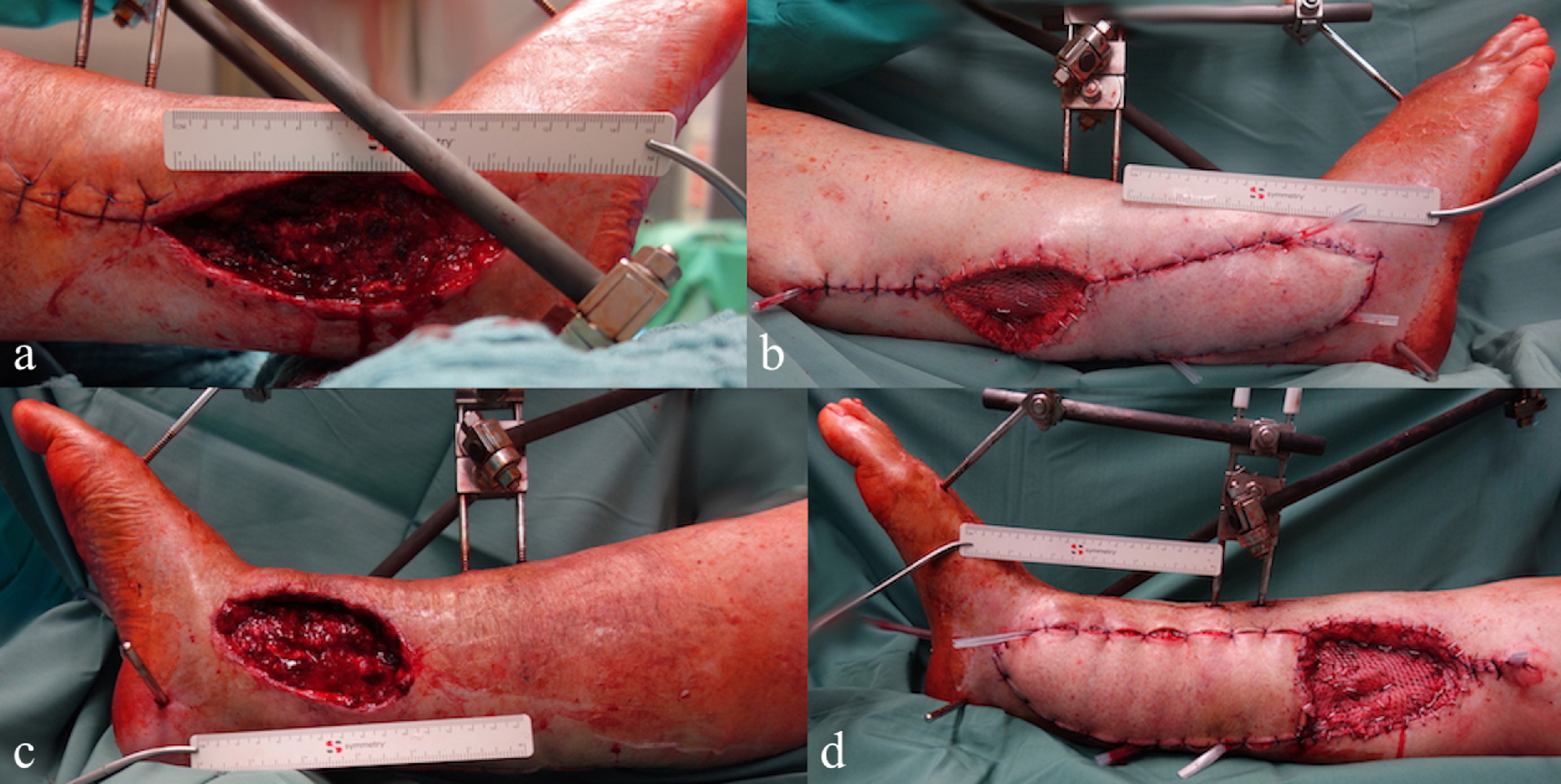

Supplement: Supplementary file 2 — Figure 3. View on lateral defect (a) and after reconstruction with propeller flap (b); view on medial defect (c) and after reconstruction with propeller flap (d). [file mmc2.jpg]

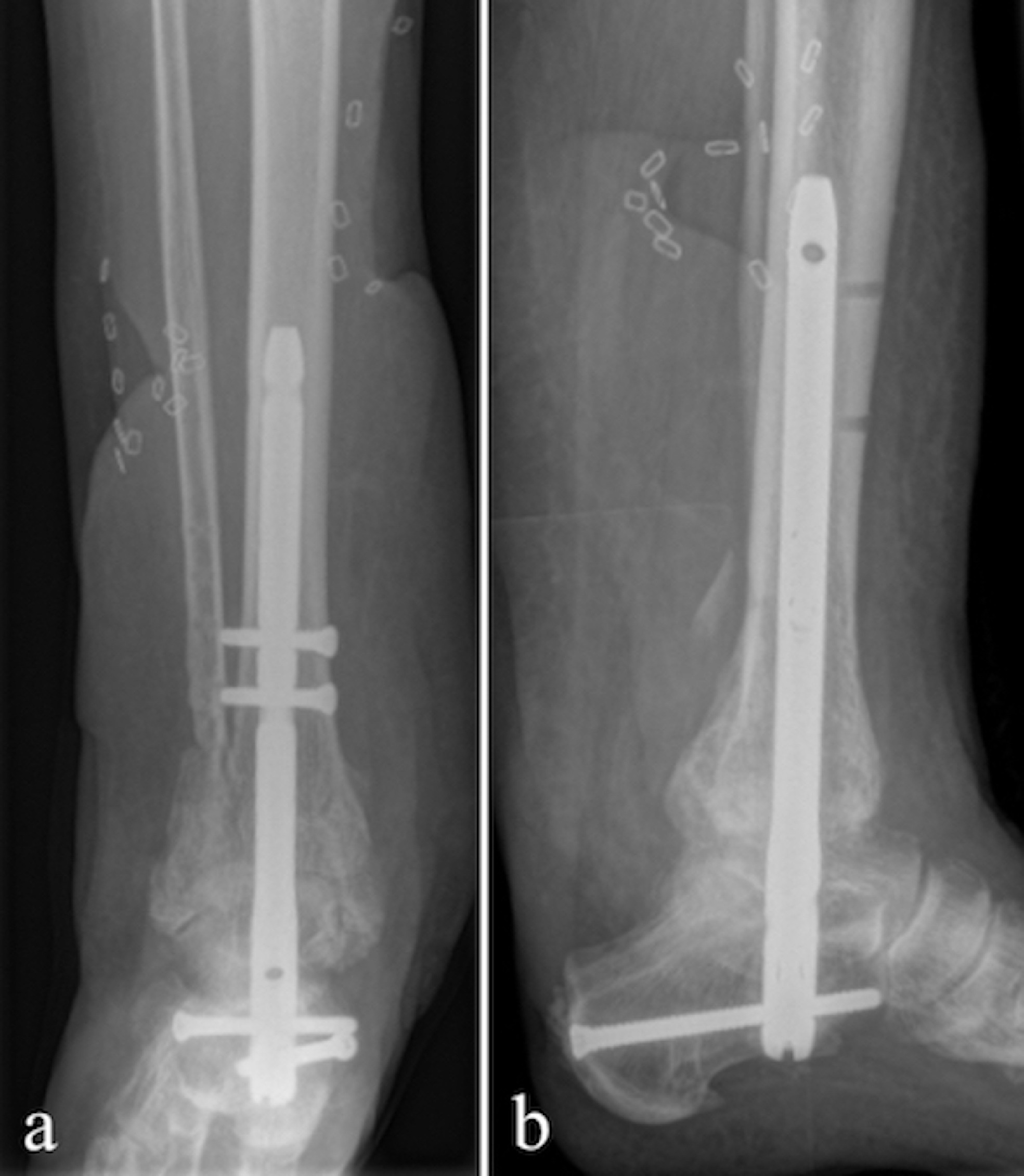

Supplement: Supplementary file 3 — Figure 4. Radiological findings after arthrodesis with retrograde intra-medullar nail (Valor™, Hindfoot Fusion Nail System, Wright Medical Group N.V.): anterior view (a), lateral view (b). [file mmc3.jpg]
